# Morphological and Advanced Imaging of Epilepsy: Beyond the Basics

**DOI:** 10.3390/children6030043

**Published:** 2019-03-11

**Authors:** Aikaterini Fitsiori, Shivaprakash Basavanthaiah Hiremath, José Boto, Valentina Garibotto, Maria Isabel Vargas

**Affiliations:** 1 Unit of Neurodiagnostic, Division of Neuroradiology, Geneva University Hospital, rue Gabrielle-Perret-Gentil 4, 1205 Geneva, Switzerland; shivaprakashbh@gmail.com (S.B.H.); Jose.M.BaiaoBoto@hcuge.ch (J.B.); Maria.I.Vargas@hcuge.ch (M.I.V.); 2 Division of Nuclear Medicine and Molecular Imaging, Geneva University Hospital and Faculty of Medicine, Geneva University, 1205 Geneva, Switzerland; Valentina.Garibotto@hcuge.ch

**Keywords:** epilepsy, magnetic resonance imaging (MRI), positron emission topography-computed tomography (PET-CT), single positron emission computed tomography (SPECT), hippocampal sclerosis, cortical malformations, diffusion tensor imaging, spectroscopy

## Abstract

The etiology of epilepsy is variable and sometimes multifactorial. Clinical course and response to treatment largely depend on the precise etiology of the seizures. Along with the electroencephalogram (EEG), neuroimaging techniques, in particular, magnetic resonance imaging (MRI), are the most important tools for determining the possible etiology of epilepsy. Over the last few years, there have been many developments in data acquisition and analysis for both morphological and functional neuroimaging of people suffering from this condition. These innovations have increased the detection of underlying structural pathologies, which have till recently been classified as “cryptogenic” epilepsy. Cryptogenic epilepsy is often refractory to anti-epileptic drug treatment. In drug-resistant patients with structural or consistent functional lesions related to the epilepsy syndrome, surgery is the only treatment that can offer a seizure-free outcome. The pre-operative detection of the underlying structural condition increases the odds of successful surgical treatment of pharmacoresistant epilepsy. This article provides a comprehensive overview of neuroimaging techniques in epilepsy, highlighting recent advances and innovations and summarizes frequent etiologies of epilepsy in order to improve the diagnosis and management of patients suffering from seizures, especially young patients and children.

## 1. Introduction

Humans have long suffered from epilepsy, the neurological disorder hallmarked by sudden seizures. The term epilepsy holds its origin in the Greek word epilepsis “epilepsy”, literally “a seizure”, from epilambanein “to lay hold of, seize upon, attack” [1], which describes very well the sudden and frequently frightening presentation of this disorder. Therefore, epilepsy has for a long time been considered as a punishment of divine origin and it is referred to as the “sacred disease” in ancient texts, while during the Middle Ages it is referred to as “the falling sickness”, beautifully described in Shakespeare’s Julius Caesar [2]. Although Hippocrates was the first to put in doubt its divine origin [2], a real explosion in our knowledge on the disease’s pathophysiology, as well as in its diagnostic workup and therapeutic approach was only observed during the last century.

Nevertheless, the precise etiology of a convulsive syndrome still remains a diagnostic challenge and, for this reason, a new era in the field of epilepsy has begun with the introduction of magnetic resonance imaging (MRI) during the last few decades. Recent developments have allowed new possibilities in epilepsy neuroimaging with more powerful MRI machines, as well as the development of advanced sequences for a detailed study of the brain anatomy and pathologies. An estimated 10% to 40% of children with epilepsy have medically refractory epilepsy [3]; in many of them, an underlying structural or functional epileptogenic lesion is the cause. The search for an underlying etiology in cases of drug-resistant epilepsy is of vital importance because it may guide surgical treatment and thereby allay some of the negative psychological, cognitive and socioeconomic consequences of persistent seizures [3], especially in seizures with presentation during childhood. Therefore, there is an increased interest among researchers in investigation of newly acquired tools such as machine learning and automated methods [4,5,6,7,8], as well as a multidisciplinary approach combining electroencephalography (EEG) and MRI findings [9,10,11], with nuclear medicine techniques and genetics/molecular biology’s analysis [12,13,14,15].

In this article, we aim to address morphological imaging including new sequences with morphometric analysis. Advanced sequences and methods such as diffusion tensor imaging (DTI), spectroscopy and isotropic volumetric T1-weighted sequences are also discussed, as well as the contribution of metabolic imaging such as positron emission tomography-computed tomography (PET-CT), positron emission tomography-magnetic resonance imaging (PET-MRI) and single photon emission computed tomography (SPECT) to epilepsy. Different causes of epilepsy are illustrated with clinical cases, while on a more practical point, an optimized imaging protocol is proposed. Finally, we will shed some light on the most frequent artifacts and pitfalls in epilepsy imaging. This article summarizes a comprehensive overview of recent advances in neuroimaging of epilepsy, in order to improve diagnosis and management of patients suffering from seizures, especially young patients and children.

## 2. Morphological Imaging in Epilepsy

The introduction of MRI in the diagnostic armamentarium of epilepsy has been a revolution in the field of epilepsy and MRI is nowadays considered as the method of choice for the detection of structural lesions in chronic focal epilepsy [16]. MRI has proved more sensitive than CT for this purpose due to its higher resolution and its better gray/white matter delineation [17]; however, CT does maintain a supplementary role under certain circumstances [3], such as for detection of blood or calcifications, as those seen in children with a history of congenital infection [18], tumors with predisposition to hemorrhage or calcification, including dysembryoplastic neuroepithelial tumors (DNET), gangliogliomas or oligodendrogliomas, all common causes of refractory epilepsy. Another indication for CT imaging is the detection of bone remodeling (DNET), calcified nodules in Tuberosis Sclerosis Complex (TBC) [19], as well as in neurocysticercosis, the most common etiology for symptomatic epilepsy in much of the developing world [3,20,21]. CT is still frequently used as an emergency study at the first presentation of a convulsive syndrome, in order to rule out serious pathology that could require urgent treatment, because of its wide availability, as well as its quick acquisition, without the need for sedation, which is an important factor especially in children’s imaging [3,18]. According to the Commission on Neuroimaging of the International League Against Epilepsy (ILAE), all patients with epilepsy should undergo an MRI, except those with very typical forms of primary generalized epilepsy or benign focal epilepsies of childhood with characteristic clinical and EEG features and adequate response to antiepileptic drugs. In the next sections, we discuss some technical considerations and optimal MRI protocol for this group of patients, including basic and advanced techniques [18,22].

### 2.1. Technical Considerations

Despite recent advances, focal cortical dysplasia was found on histopathology in approximately 50% of patients with cryptogenic focal epilepsy (negative MRI finding prior to surgery) [23,24], while according to other studies, MRI successfully detected underlying abnormalities in 75% of patients with refractory focal epilepsy [23,25,26]. According to Wellmer et al., possible reasons for undetected epileptic lesions in standard outpatient MRI included: (1) insufficient clinical focus hypotheses from the referring neurologists; (2) “standard head” protocols not optimized for the spectrum of epileptogenic lesions; and (3) unfamiliarity with the spectrum of epileptogenic lesions [27].

Therefore, a meticulous examination of MRI images by the interpreting radiologist is essential for the detection of subtle abnormalities, with a particular focus on areas highlighted by clinical and/or EEG findings. A close correlation with clinical and EEG findings is also necessary in order to determine whether an identified lesion is likely to be the cause of the seizures because some frequent MRI findings such as encephalomalacia or arachnoid cysts can be incidental and asymptomatic [3]. Several studies have demonstrated that the detection of the epileptogenic lesion depends on the expertise of the radiologist who interprets the MRI study [22,26,28]; for this reason, some authors suggest that all patients with refractory epilepsy and a “nonlesional” routine MRI should be referred to tertiary centers with expertise in epilepsy [17,26] and should be evaluated by a multidisciplinary team, including neurologists specialized in epilepsy, neuroradiologists and neurosurgeons.

In addition to the fact that the interpreting radiologist should be familiar with epilepsy, a dedicated MRI protocol is also required for improved lesion detection, according to Spencer D. [29] (39% detection rate with “standard head” MRI protocol interpreted by a “non-expert” radiologist against 91% with optimized for epilepsy protocol and specialized neuroradiologist). A “standard head” MRI protocol, including T1 and T2-weighted sequences, is able to reveal some epileptogenic lesions such as tumors or encephalomalacia but may miss subtle abnormalities such as focal cortical dysplasia (FCD). An optimized epilepsy protocol is discussed in the next section.

Three Tesla (3T) MRI increases the detection of epileptogenic lesions (2.57 times more likely than 1.5 Tesla according to Phal et al. [30]). This has been demonstrated by several studies using 3 Tesla (3T) MRI machines compared to 1.5 Tesla (1.5T) [31,32], while other studies suggest ultra-high field 7 Tesla (7T) MRI is superior to 3T MRI machines [33,34]. Higher field MRI performed better not only in the detection but also in characterizing identified lesions [23,30]. Increased image quality with higher signal-to-noise ratio and increased spatial and contrast resolution were also achieved—according to some studies—with the use of multichannel phased-array surface coils compared to conventional quadrature head coil imaging, which has been used in the past [35,36].

### 2.2. Conventional MR Imaging

According to the recommendations of the ILAE, all epilepsy protocols should include thin-sliced volumetric T1-weighted gradient-recalled echo sequence, axial and coronal T2-weighted sequences and high-resolution oblique coronal hippocampal, axial, and sagittal fluid-attenuated inversion recovery (FLAIR) sequence(s) or a three-dimensional FLAIR sequence [18]. Similarly, Wellmer et al. proposed an “essential 6” sequence protocol allowing, according to their study, the detection of virtually all common epileptogenic lesions. The 6 components of their protocol included: a 3D isotropic T1 sequence, at least two planes of T2 and FLAIR acquisition and a hemosiderin/calcification sensible sequence [27]. Skjei et al. proposed additional sequences, such as T1 Inversion Recovery coronal images, as well as serial imaging in younger children (less than 2 years of age) because of immature myelination [3]. Gadolinium contrast was proposed only in cases of suspected tumor, vascular malformations, or infection/inflammation or if indicated based on the findings on non-contrast imaging [3].

High resolution coronal T2 images (2–3 mm thickness) and an acquisition plane perpendicular to the long axis of the hippocampus is complementary to axial T2 acquisition and allow improved detection of hippocampus abnormalities, such as hippocampal sclerosis (HS), a common etiology of refractory epilepsy in children and adults, as well as detection of temporal encephaloceles, often missed in other planes or sequences [37].

FLAIR can enhance T2 conspicuity of epileptogenic hyperintense lesions, such as HS, tumors, gliotic scars or FCD [23,38,39,40,41,42]. Depending on sulcal orientation, coronal, sagittal or axial images can be useful in the search of subtle cortical abnormalities and, for this reason, in many institutions a 3D isotropic (1 mm thickness) FLAIR sequence is performed in routine epilepsy screening [36,43,44]. Post-processing is also available for 3D FLAIR images with automated or semi-automated detection of lesions [44,45] and can be useful in some cases.

Sequences sensitive to the presence of hemosiderin/calcification such as T2*-weighted gradient-recalled echo (GRE) or susceptibility weighted imaging (SWI) demonstrate calcifications and hemorrhage and increase sensitivity for the detection of small focal epileptogenic lesions [46], such as cavernomas or calcified tubers and nodules in TSC or neurocysticercosis. Due to susceptibility artifacts in areas near the skull base, some authors propose acquisition in the coronal plane for detecting small lesions in these areas [47].

Epilepsy imaging protocols include 3D isotropic T1-weighted sequences, which provide an excellent assessment of cortical thickness and improved delineation between gray/white matter, especially important in searching cortical malformations. For this purpose, optimized 3D T1-weighted GRE isotropic sequences (magnetization prepared rapid acquisition GRE [MPRAGE] or spoiled gradient-recalled acquisition [SPGR]) with high spatial resolution (1-mm isotropic voxels) are used [37]. Furthermore, with these sequences, a variety of post-processing techniques and analyses is possible, such as multiplanar and curvilinear reconstruction, both with an important diagnostic application in epilepsy ([47,48] respectively). In addition, volumetric acquisition sequences can also be used to perform hippocampal segmentation and volume measurements [37,49], as well as for automated lesion detection with different post-processing techniques [50].

### 2.3. Advanced Imaging: Beyond the Basics

Diffusion tensor imaging (DTI) is a relatively new MRI sequence that allows tracking of white fibers in the brain and spine. Both changes in diffusion (or associated apparent diffusion coefficient-ADC maps) and/or DTI were described in cases of otherwise “normal” MRI study and thought to be helpful in identifying epileptogenic zones either for surgical treatment or for placing intracerebral electrodes for stereo-electroencephalography (SEEG) [51,52]. Histopathological studies have demonstrated subtle changes in areas with DTI abnormalities that were not visible in other sequences [53,54], while reduced anisotropy and increased diffusivity have been demonstrated in the white matter subjacent to the dysplastic cortex [37]. Furthermore, diffusion can demonstrate transient MR signal changes that occur after generalized tonicoclonic seizure or status epilepticus in the form of increased signal at the cortical gray matter, subcortical white matter, or hippocampus in the periictal period [55]. These findings reflect transient cytotoxic and vasogenic edema induced by seizures [55].

**Figure 1:**
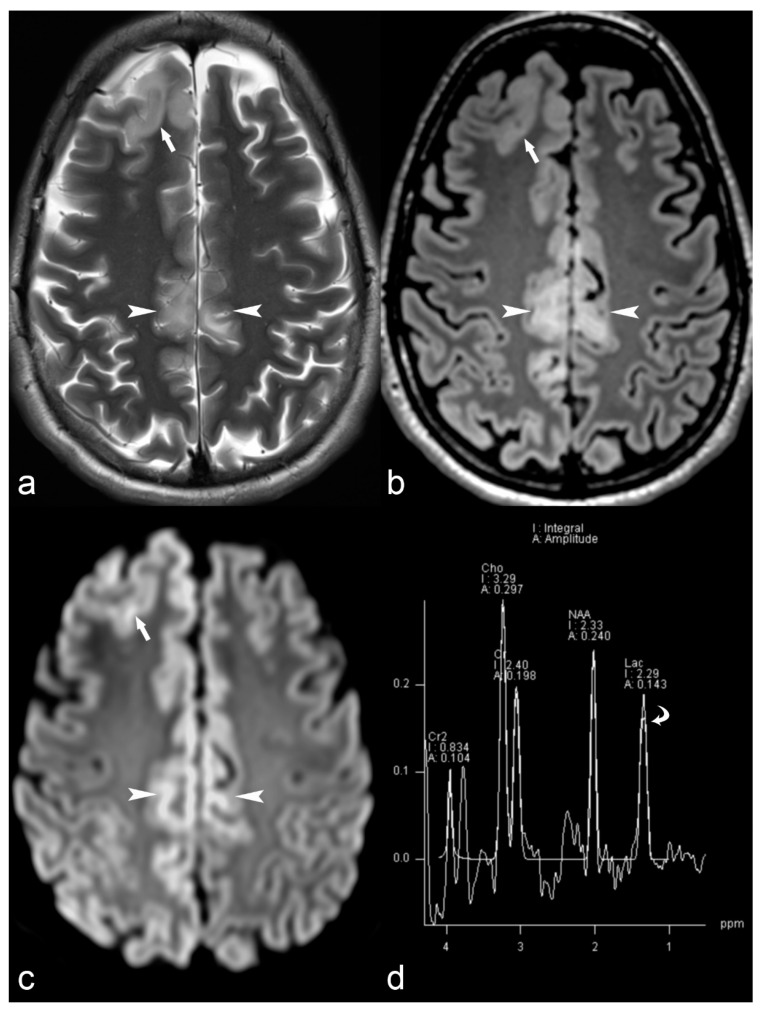
33-year-old female with MERRF (myoclonic epilepsy with ragged red fibers) and myoclonic epilepsy. Axial T2 (**a**), Fluid-attenuated inversion recovery (FLAIR) (**b**) and Diffusion weighted imaging (DWI) (**c**) trace images (b = 1000) show cortical thickening in the right frontal (arrow) and bilateral pericingulate region (arrowhead) with subcortical white matter hyperintensity and restricted diffusion (ADC images not shown). Magnetic Resonance (MR) spectrum (**d**) at short TE (35 ms) at the level of right pericingulate lesion (region of interest of 1 cm diameter) shows lactate peak at 1.33 ppm (curved arrow) consistent with mitochondrial encephalopathy.

Magnetic resonance spectroscopy is an MRI technique that provides biochemical information on selective areas of the brain and can play an adjunctive role in the presurgical evaluation of certain patients with drug-resistant epilepsy. Different techniques including monovoxel or multivoxel studies have been used for the characterization of brain tumors, as well as for other pathologies, including leucodystrophies, metabolic disorders (Figure 1) or HS. MRS can also be useful in identifying the side of the epileptogenic focus in cases of temporal lobe epilepsy, demonstrating decreased N-acetyl aspartate (NAA) to creatine (Cr) ratio or NAA to choline (Cho) ratio in the pathologic site [56,57], frequently before morphological changes become apparent in conventional imaging.

Magnetoencephalography is an advanced method used to measure magnetic fields generated by small intracellular neuronal electrical currents [37] in order to localize ictal and interictal spike sources. It can be useful in guiding deep electrode placement in cryptogenic forms of epilepsy, but it cannot differentiate between different types of epileptogenic lesions [3].

**Figure 2:**
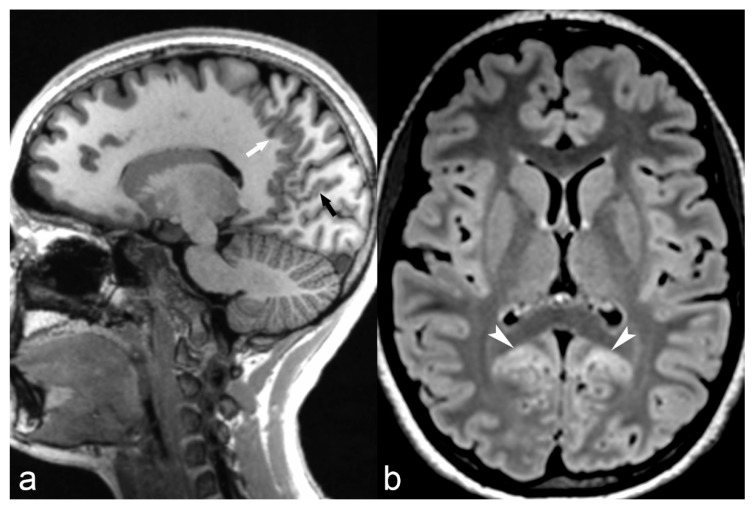
14-year-old female with parieto-occipital polymicrogyria. Sagittal T1 magnetization/prepared-2-rapid-acquisition-gradient-echo (MP2RAGE) (**a**) image showing numerous small gyri involving the parasagittal parieto-occipital region (arrow) and calcarine sulcus (black arrow) with mild cortical hyperintensity (arrowheads) on axial FLAIR (**b**) images suggestive of polymicrogyria.

The magnetization-prepared two rapid acquisition gradient echoes (MP2RAGE) sequence is a volumetric T1 weighted novel sequence, which acquires two gradient echo images after an inversion pulse and is currently used as part of the routine epilepsy protocol in our institution. It provides excellent contrast-to-noise ratio with minimal effect of B1 inhomogeneity (B1 is the radiofrequency field, which is applied perpendicular to the main magnetic field of an MRI machine) and improved delineation of gray/white matter interface (well demonstrated in Figure 2a) and has, therefore, allowed increased detection and characterization of subtle cortical malformations, especially FCD [33]. Furthermore, it allows automated volume calculation of different brain regions [33].

Susceptibility-weighted imaging (SWI) is another recently developed sequence, which enhances the T2 GRE sequence sensitivity to the presence of calcium or iron products, such as deoxyhemoglobin or hemosiderin and therefore can increase the diagnostic rate of calcified epileptogenic lesions such as cortical tubers or nodules, Sturge-Weber angiomatosis, intratumoral calcifications as well as hemorrhagic lesions such as neoplasms or vascular malformations. The SWI sequence also allows distinction between calcification and hemorrhage and thus, supplementary CT for this purpose can be avoided, reducing irradiation risk, especially in the pediatric population. Furthermore, according to Pittau et al., SWI in 7T ultra-high field revealed subtle changes in the vascular architecture of the cortical lesions responsible for medically refractory epilepsy [33].

In addition to improved sequences, recent advances in epilepsy neuroimaging include automated or semi-automated algorithms for segmentation, morphometric analysis and volumetry of different brain regions, which can be a useful tool for early diagnosis of HS, FCD or TSC [58,59,60]. For this purpose, different computing programs and algorithms have been proposed by different authors including freesurfer [59] and multi-atlas–based segmentation propagation method [58] with various outcomes. According to House et al., morphometric analysis in patients with TSC increased tuber detection [60]. There are several studies demonstrating interesting results with automated volume calculation or lesion detection based on 3D T1 and FLAIR sequences [37,45,49,58,59,60].

Finally, functional MRI for the detection of eloquent areas of the brain and their proximity to epileptogenic lesions, currently plays an important role in everyday practice in many centers as part of preoperative studies, in order to adequately guide surgical resection with the least damage to brain functions. Other promising functional MRI paradigms assess memory, visual and somatosensory pathways, while simultaneous recording of EEG and functional MRI can provide information in networks underlying seizure generation [61].

A proposition for an optimized MRI protocol for diagnosis of focal epilepsy, as currently performed in our institution, is summarized in Table 1.

## 3. Metabolic Imaging (PET-CT, PET-MRI, SPECT) in Epilepsy

Nuclear medicine techniques provide important functional information for the presurgical evaluation of pharmacoresistant epileptic patients, measuring in vivo perfusion changes associated with the seizure, metabolic changes and neurotransmission abnormalities [22].

**Figure 3:**
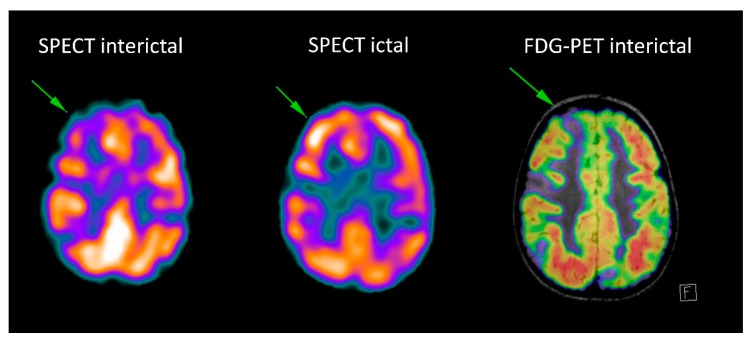
Patient with tuberosis sclerosis complex: the interictal Single-photon emission computed tomography (SPECT) and fluorodeoxyglucose-positron emission tomography (FDG-PET) (fused with MRI FLAIR images) show multiple hypometabolic foci, corresponding to the multiple tubers. Ictal SPECT shows ictal hyperperfusion of the superior right frontal tuber.

The most commonly used imaging strategy is 18F-fluorodeoxyglucose positron emission tomography (FDG-PET) in the interictal state, allowing detection of focal areas of relative hypometabolism, that are presumed to reflect focal functional disturbances of cerebral activity associated with the epileptogenic tissue, as exemplified in Figure 3 [62,63]. The interpretation of FDG-PET images with the support of automated semiquantitative approaches and with a systematic fusion of PET and MRI has a relevant diagnostic added value, namely for neocortical epilepsy [64]. For this purpose, technological developments, namely the availability of new hybrid tomographs allowing PET and MRI imaging in a single session, provide an ideal tool for a comprehensive investigation of epileptic patients [65,66]. Recent studies have also suggested that focal FDG-PET abnormalities carry not only diagnostic but also prognostic information and are a predictive factor for a positive surgical outcome, both in temporal and extratemporal epilepsy [64]. Finally, PET imaging using specific molecular tracers also allows the assessment of various neurotransmission systems, with promising results in clinical research applications [67].

Ictal SPECT with perfusion tracers is the only technique able to show the perfusion changes occurring during the epileptic event: two acquisitions are usually performed, in the ictal and interictal condition (as exemplified in Figure 3) and digital subtraction techniques are used to complement visual interpretation to identify the subtlest changes associated with the seizure.

## 4. Frequent Etiologies of Refractory Epilepsy

### 4.1. Hippocampal Sclerosis

**Figure 4:**
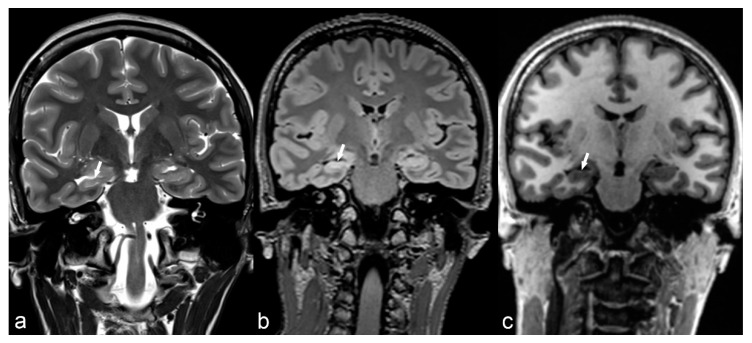
36-year-old woman. The right hippocampus is smaller than the left (arrows) as seen on coronal T2WI (**a**), FLAIR (**b**) and MP2RAGE (**c**) and shows hyperintensity on T2WI and FLAIR. There is also flattening and loss of the normal undulations of the right hippocampus, suggestive of hippocampal sclerosis.

Hippocampal sclerosis is by far the most frequent etiology of temporal lobe epilepsy, present in approximately 60%–80% of histopathological studies after temporal lobe resection for epilepsy [68]. It is usually a disease of older children and young adults [69] and it is characterized by gliosis combined with atrophy and loss of the internal architecture of the involved hippocampus. In conventional studies, characteristic findings include hippocampal atrophy and T2/FLAIR hyperintensity (Figure 4). Additional findings in advanced sequences may be present such as atrophy of other parts of the ipsilateral limbic system, including the amygdala, mamillary body or entorhinal cortex [37]. On histopathology, HS is characterized by neuronal loss with gliosis [23,70]. HS can be unilateral or bilateral, symmetrical or asymmetrical. If bilateral, diagnosis can be challenging and, for this reason, advanced techniques including spectroscopy, quantitative techniques for volume calculation or T2 intensity and metabolic imaging have been used, along with conventional T1, T2 and FLAIR images, especially in the coronal plane [37,50,56,57]. An interesting finding observed in 32% to 66% of adults with HS [71,72], as well as in the pediatric population with a similar prevalence [72], is T2 hyperintensity of the anterior temporal lobe with blurring of gray/white matter junction; this finding is usually related to early onset of seizures, thought to represent persistent immature appearance and can be misdiagnosed as FCD [72]. Other pathologies such as low-grade tumors, cortical malformations, vascular malformations or ischemic insults may coexist with HS, frequently referred to as “dual pathology” [73]. Surgical removal of the involved hippocampus in cases of unilateral disease can achieve a seizure-free outcome in approximately 80% [74] of patients, while in bilateral disease there is a poorer outcome and increased risk for neuropsychological decline.

### 4.2. Focal Cortical Dysplasia

**Figure 5:**
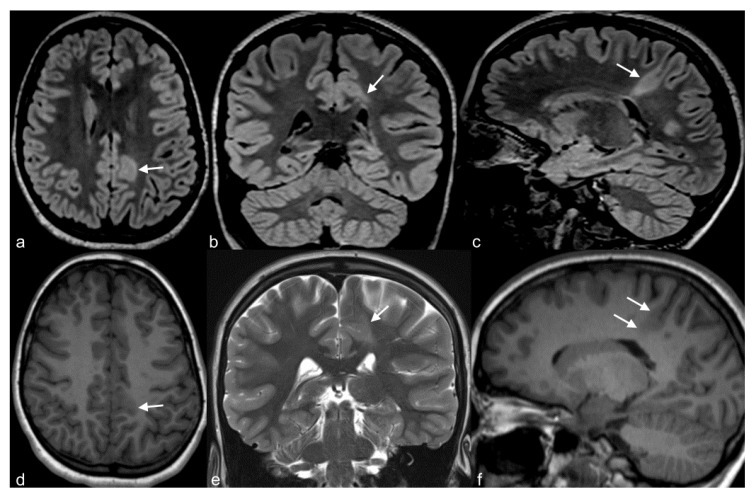
10-year-old girl with cortical dysplasia. There is thickening of the cortex on the left mesial fronto-parietal region (arrows in (**a**) to (**f**)) associated to funnel-shaped hyperintensity of the surrounding white matter (arrows in (**b**,**e**)). Note blurring between the white and gray matter interface (**f**).

Focal cortical dysplasia (FCD) is probably the most common cause of refractory extratemporal focal epilepsy, especially in the pediatric population [75]. Epilepsy due to FCD commonly begins in the first few years of life and may occur shortly after birth [75]. The term “focal cortical dysplasia” was introduced by Taylor et al. [76] in order to describe a malformation of cortical development in the human brain that consisted of disorganized cortex with enlarged dysplastic neurons and enlarged balloon cells. This type of FCD is nowadays classified as FCD type II; type I FCD is also characterized by abnormal cortex with distorted architecture, but individual neurons are normal and not enlarged as in type II [77]. A type III FCD has recently been described, but it is not generally considered a distinct entity in itself; it is FCD I or II associated with another primary lesion, such as HS, tumor, vascular malformation, ischemic scars, or other [78]. FCD type II is characterized by focal cortical thickening, “blurring” of gray/white matter interface, subcortical T2/FLAIR hyperintensity and sometimes a funnel-shaped high T2/FLAIR signal pointing towards the ventricles (“transmantle sign”) (Figure 5) [76,77,78], while findings in FCD type I are usually subtler and this latter is usually more difficult to detect. FCD has a predilection for the frontal lobes, contrary to HS and epileptogenic tumors, usually located in the temporal lobes. FCD represents a great part of “nonlesional” MRIs in patients with medically refractory epilepsy, due to its small size or subtle architectural changes, undetected by neuroimaging techniques. For this reason, multiplanar reconstruction of high-resolution images, as well as advanced methods such as DTI and spectroscopy are important for detection and confirmation of subtle lesions, especially when these are located at the bottom of the sulcus. DTI shows decreased connectivity in the area around the dysplastic cortex and spectroscopy shows decreased NAA/Cr ratio. These techniques or metabolic imaging (PET-CT and SPECT imaging) may highlight suspicious cortical areas, that can be re-inspected on initial MR images [23,79] and increase lesion detection this way. Complete resection of FCD is the best predictor for seizure control [23,42] and for this reason surgical treatment has better outcomes for FCD type II compared to type I. Presurgical detection of the epileptogenic focus is therefore of the utmost importance. Close correlation of semiology, EEG, MRI with advanced techniques and/or ultra-high field, as well as PET-CT/SPECT findings, is required in order to improve prognosis not only for seizure control of these patients, but also for the developmental outcome in the pediatric population suffering from epilepsy due to FCD treated by surgery at early stages [23,80,81].

### 4.3. Other Cortical Malformations

Although not all patients with malformations of cortical development present with epilepsy [82], there is a spectrum of cortical malformations associated with refractory epilepsy, including FCD, polymicrogyria, lissencephaly/pachygyria, schizencephaly, megalencephaly and heterotopia [83]. They usually result from an error in one or more of the orderly processes of neuroblast proliferation and differentiation, neuroblast migration and cortical organization during in utero development of the human brain or may be genetically determined and inherited [83].

Polymicrogyria is characterized by an excessive number of small convolutions associated with shallow sulci, giving the surface of the brain its characteristic “lumpy appearance”. It can be unilateral or bilateral, symmetrical or asymmetrical and may be associated with epilepsy, developmental delay/cognitive impairment or focal neurologic deficits [83]. The age of presentation varies with the extent and/or the location of the malformation [69], but as for most of the other cortical malformations a presentation with seizures during childhood or in young adults is a common feature. It has a predilection for the perisylvian area, while other common locations are the frontal, parietal and occipital lobes. MRI studies demonstrate multiple small gyri (Figure 2) and an indistinct gray and white matter junction [69]. The thickness of the cortex may be normal or abnormally thick or thin [84], but if imaging is acquired with thick slices and low spatial resolution it can mimic a pachygyric pattern [82]. Polymicrogyria is often associated with other malformations such as corpus callosum agenesis, cerebellar hypoplasia and gray matter heterotopia [82].

Schizencephaly describes a cleft extending from the cerebral cortex to the ventricular surface and at its edges is usually lined by the polymicrogyric cortex. Schizencephaly also has a predilection for the perisylvian cortex and insula. We can distinguish two types: the “closed-lip” type, with small defects that are fused and the “open-lip” type with large defects which are filled with cerebrospinal fluid [84]. Clinical manifestations include seizures, developmental delay, spasticity and paresis and are more severe in the “open-lip” type [84]. They are distinguished by porencephaly in that the clefts are lined by abnormal gray matter, typically polymicrogyric cortex [85]. A characteristic MRI finding is a “dimple” outwards from the lateral wall of the lateral ventricle indicating the site where the cleft reaches the ventricle [83]. The microgyric pattern lining the cleft, with gray matter signal intensity in classical sequences helps differentiation from the high T2 signal of gliotic changes or white matter, like those seen in porencephaly. Routine MRI sequences are usually sufficient for the diagnosis of this disorder, especially for the “open-lip” schizencephaly, where spaces filled with fluid are well demonstrated between the brain surface and the ventricles. Conspicuity of the lesions and evaluation of their extension can be facilitated by multiplanar and curvilinear reformats [83]. Associated findings include septum pellucidum agenesis, corpus callosum dysgenesis and optic nerves hypoplasia. For this reason, some cases of schizencephaly are considered part of the septo-optic dysplasia spectrum [85]. There is evidence that CMV in utero infection, ischemic insults and genetic causes contribute to the pathogenesis of the disorder [85] and common presentations include “congenital” hemiparesis or early-life seizures, as well as developmental delay and spasticity [69].

**Figure 6:**
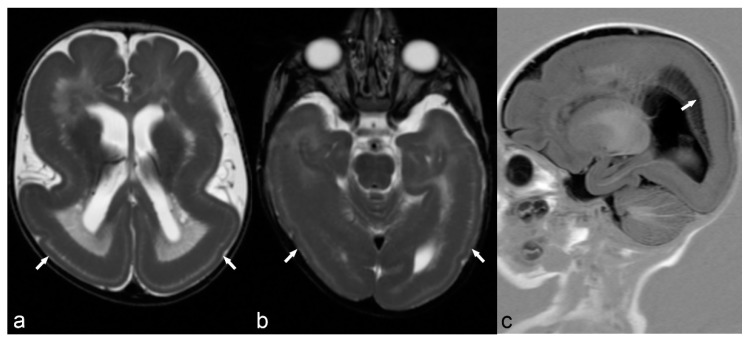
6-month-old baby. Axial T2WI slices through the lateral ventricles (**a**) and occipital lobes (**b**) demonstrate a distinct lack of normal brain gyration with a smooth and thickened appearance of the cortex and unfolded gyri, most pronounced in both parietal, temporal and occipital lobes (arrows) consistent with lissencephaly. Note the posterior-anterior gradient with some rudimental sulcation seen in the frontal lobes. Band heterotopia can be clearly seen in the parietal regions on sagittal T1 IR (**c**) with cobblestone appearance (arrow).

Classical lissencephaly is characterized by reduced or absent gyri associated with a thickened cortex and shallow sulci. Microscopically, the cortex lacks the normal lamination and consists of only 4 layers instead of the typical 6 layers. Macroscopically, the sylvian and Rolandic fissures are poorly developed and there is a failure of operculization of the insular areas [85]. This results in a smooth appearance of the brain surface with the characteristic “Figure 8” or “hourglass” shape of the brain in axial images (Figure 6) [83]. There are several gene mutations implicated in its pathogenesis and a wide spectrum of clinical manifestations such as severe intellectual disability, hypotonia and epilepsy. It is usually diagnosed early in life, although mild cases may have delayed presentation [69]. Miller-Dieker syndrome (MDS) is a severe form of classical lissencephaly associated with facial dysmorphism and occasionally other congenital abnormalities, epilepsy and a severely shortened life expectancy, related to a chromosome 17 gene deletion. Cobblestone lissencephaly is a distinct form of lissencephaly characterized by a nodular appearance of the brain cortex secondary to abnormal organization of the cortical layers.

Hemimegalencephaly is a severe malformation involving one hemisphere or part of it and characterized by overgrowth of the hemisphere, with dysplastic, often thickened, cortex, and an abnormal signal of white matter. It is usually diagnosed during the 1st year of life [69]. MRI demonstrates enlargement of the hemisphere, abnormal T1 and T2 signal of white matter, and often enlarged ipsilateral ventricle with pointed frontal horn [86]. Association with FCD, polymicrogyria, pachygyria or heterotopia is not uncommon [82].

Heterotopia can be either nodular or laminar (band). It results from an insult during neuronal migration from the subependymal matrix to the cortex. Severe cases present in infancy with seizures and severe motor and cognitive dysfunction, while milder cases may appear during the 2nd decade of life with epilepsy [69]. Its main characteristic is that lesions follow the signal of gray matter in all sequences. Subependymal or subcortical nodular heterotopia appears as nodules of gray matter adjacent or protruding into the ventricles or within subcortical white matter, while subcortical band heterotopia has the characteristic appearance of “double cortex”, with a four-layer appearance of the brain [82,83]. Nodules may be unilateral or bilateral, unique or multiple, small or large. Subcortical band heterotopia has a clear female predominance and can be either sporadic or familial. Several gene mutations have been identified as responsible for this disorder and associated malformations of the brain are also commonly present [85].

### 4.4. Neurocutaneous Syndromes

**Figure 7:**
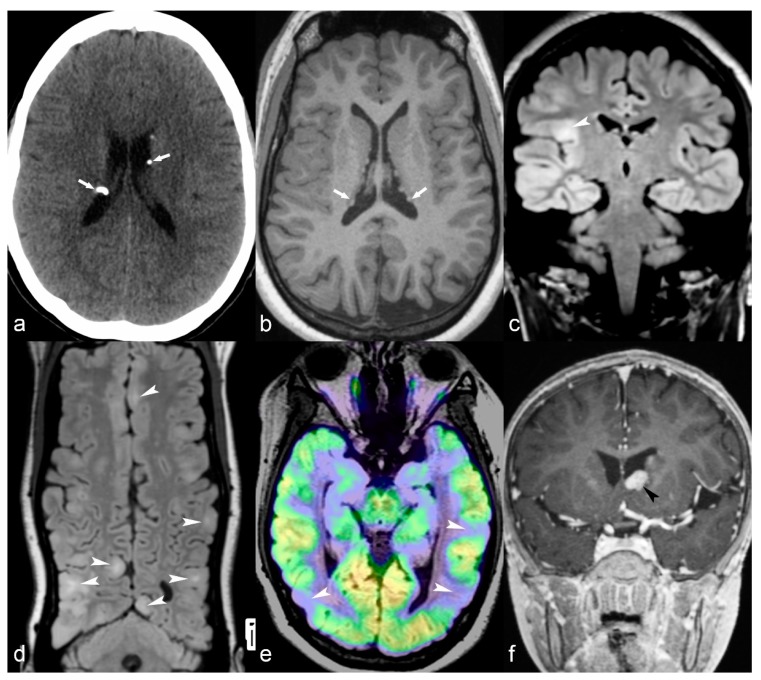
(**a**) 25-year-old female with Tuberosis Sclerosis Complex (TSC). Axial CT image (**a**) shows multiple calcified subependymal nodules (arrows). (**b**–**e**) Another 23-year-old female with TSC. Axial T1 weighted image (**b**) showing multiple subependymal hyperintense nodules (arrows). Coronal FLAIR (**c**) with curvilinear multiplanar reformat (**d**) shows multiple cortical tubers with radiating subcortical white matter hyperintensity (arrowheads). PET CT (**e**) shows hypometabolic areas corresponding to cortical tubers in bilateral occipito-temporal and left temporal region (arrows). (**f**) 14-year-old male with TSC. Coronal reformat of 3D T1 post contrast image (black arrowhead) shows enhancing subependymal giant cell astrocytoma abutting the floor of frontal horn of left lateral ventricle.

Tuberosis sclerosis complex (TSC) is a neurocutaneous syndrome usually involving the brain, skin, eyes, heart and kidneys. Brain lesions include cortical tubers, subependymal nodules, abnormal signal of white matter and subependymal giant cell astrocytomas [83]. There are different degrees of severity and clinical manifestations, but epilepsy is a common finding and usually consists of partial seizures originating in cortical tubers with presentation shortly after birth [85]. In other cases, it can be presented later in children with seizures, autistic-like behavior and mental retardation [69]. MRI readily demonstrates cortical tubers with high T2/FLAIR signal, often focally expanding the cortex (Figure 7). They usually do not enhance, but there is evidence that in case of multiple involvement, the largest tuber (usually the one that shows enhancement and hypermetabolic activity in perfusion MRI studies or PET-CT) is likely the one responsible for the seizures. Subependymal giant cell astrocytomas are grade I WHO tumors, can show enhancement and are usually located in proximity to the foramen of Monro in the lateral ventricles (Figure 7f). Subependymal nodules are periventricular or may protrude into the ventricles, may enhance or may be calcified, showing loss of signal in T2 GRE or SWI sequences or high attenuation on CT images (Figure 7a). Linear hyperintensities of white matter can also be identified in MRI studies, reflecting white matter radial migration lines [69].

Sturge-Weber syndrome or encephalotrigeminal angiomatosis is a rare neurocutaneous syndrome characterized by capillary-venous malformations involving skin and brain. The syndrome classically presents with a facial port-wine nevus in the trigeminal nerve innervation regions and dural and leptomeningeal angiomatosis [22]. Seizures usually in the form of infantile spasms develop during the 1st year of life [69]. In the early stages, contrast CT and MRI demonstrate diffuse leptomeningeal enhancement of the hemisphere involved or part of it, associated with enlargement of the ipsilateral choroid plexus of the lateral ventricle. In later stages, hemiatrophy of the hemisphere involved, as well as calcification with a gyriform pattern is well demonstrated in both CT or MRI with sequences sensitive to calcium.

### 4.5. Tumors

**Figure 8:**
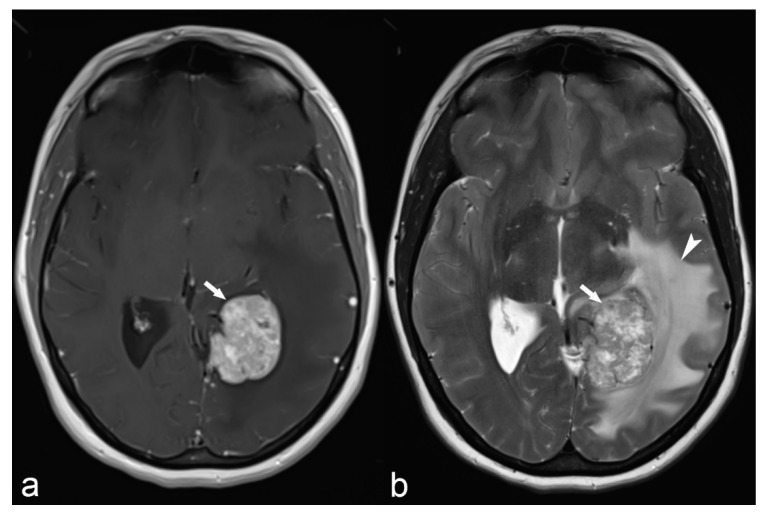
31-year-old woman. Axial T1 Gd (**a**) and T2WI (**b**) demonstrate a large meningioma arising from the left tentorium cerebelli (arrows). The mass exerts significant mass effect on the adjacent brain parenchyma with vasogenic edema (arrowhead) of the left temporal and occipital white matter.

All tumors, primary or secondary, benign or malignant, that involve the brain cortex can be potentially epileptogenic. There is, however, a propensity of some tumors to cause epilepsy such as gangliogliomas, dysembryoblastic neuroepithelial tumors (DNET), pleomorphic astrocytomas, diffuse astrocytomas, oligodendrogliomas, and some anaplastic tumors [22]. They can occur in any part of the brain, but preferentially affect the temporal lobe region [22]. Gangliogliomas are the most common tumors responsible for refractory temporal lobe epilepsy in children and young adults. MRI appearance can be variable and sometimes differential diagnosis between the different types is only possible in pathology. Common findings are cystic and nodular components, variable enhancement or calcification, cortical/subcortical location, bone scalloping or remodeling and absence of edema. They are often associated with brain malformations. Hamartoma of the tuber cinereum is a rare lesion representing a developmental malformation of the hypothalamus usually presenting with gelastic seizures between 1–3 years of age [69], or with precocious puberty. Late-onset epilepsy in elderly patients may be the first manifestation of neoplasia and in these cases, contrast administration should be considered. This is because small metastatic lesions in the cortical/subcortical junction may not be associated with significant edema and therefore only become apparent with contrast enhancement. Large extra-axial tumors, most commonly meningiomas, especially atypical ones or those associated with significant edema, can also infrequently cause seizures most commonly in older women (Figure 8).

### 4.6. Vascular Malformations

**Figure 9:**
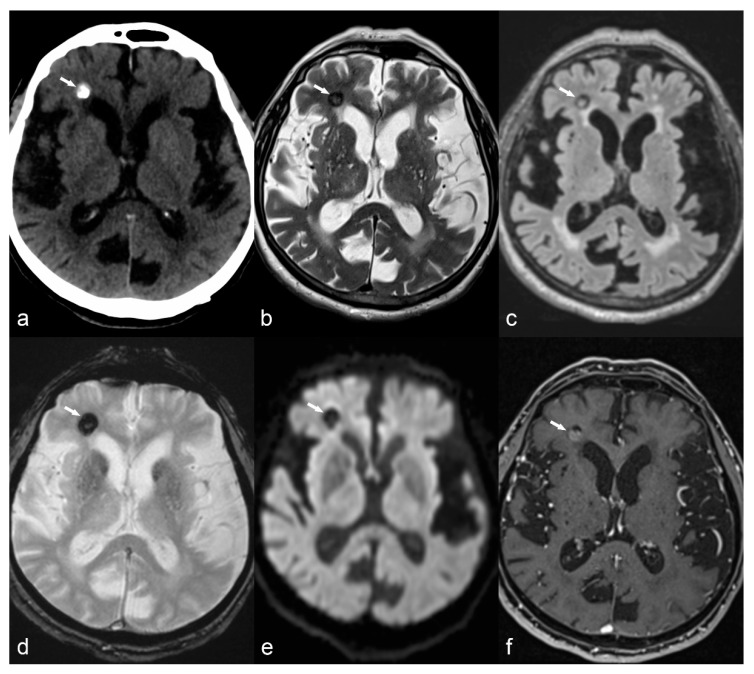
Right frontal cavernoma. Axial CT image (**a**) shows right frontal periventricular hyperdensity (arrow) abutting the frontal horn of the lateral ventricle. Axial T2 (**b**), FLAIR (**c**), GRE (**d**) and DWI (**e**) trace images (b = 1000) show peripheral hypointense rim with central hyperintensity (arrow). Axial T1 VIBE post-contrast image (**f**) shows mild enhancement (arrow) consistent with cavernoma.

There are two types of vascular malformations associated with epilepsy: cerebral cavernous malformations, or cavernomas, and arteriovenous malformations (AVM). Cavernomas represent conglomerates of abnormal vessels with a propensity for recurrent low-grade hemorrhage. Characteristic MRI findings include typical “salt and pepper” appearance on T2WI, hypointense ring on T1 or T2 images due to hemosiderin deposition, “blooming artifact” on SWI sequence and calcification seen as a low signal on MRI and high attenuation on CT (Figure 9). Arteriovenous malformations can cause epilepsy either by a direct effect to the cortex or secondary to hemosiderosis as a consequence of previous bleeding. The characteristic serpiginous appearance of dilated vessels with a nidus and often associated intranidal aneurysms is well demonstrated after contrast enhancement on CT angiography or on MR angiography with or without contrast administration. Conventional angiography still remains the method of choice for the diagnosis of AVM and may also be used as part of its treatment. Cavernomas usually present between 40–60 years of age but in some cases, they may present in childhood between 1–3 years of age [69]. AVM have a peak presentation in the 3rd to 5th decade of life, but in 25% they may present before the age of 15 between 1–3 years of age [69].

### 4.7. Gliotic Scars

**Figure 10:**
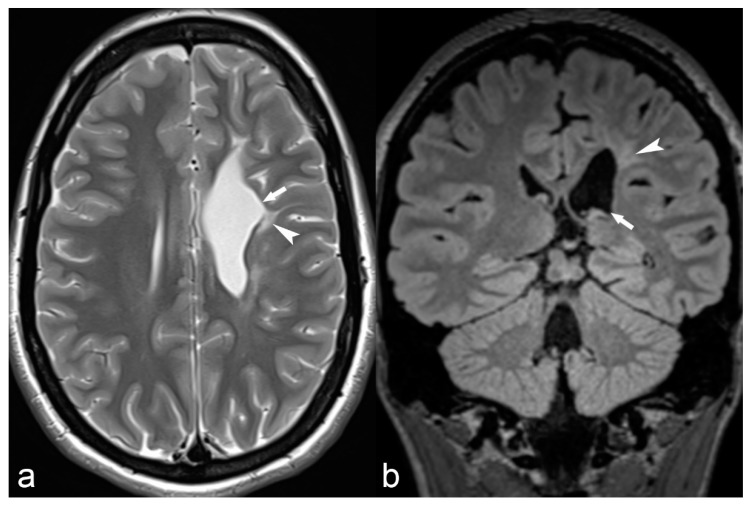
20-year-old female with epilepsy. Axial T2 weighted (**a**) and coronal FLAIR (**b**) images show irregularity of the contour of the left lateral ventricle with ex vacuo dilatation, hyperintensity and paucity of periventricular white matter (arrowhead) in the frontal region suggestive of sequalae of perinatal insult i.e., periventricular leukomalacia.

**Figure 11:**
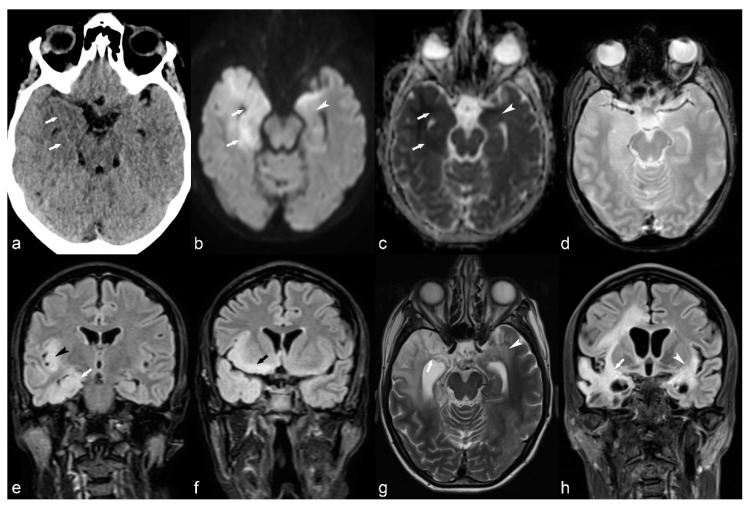
35-year-old woman with herpes encephalitis. Non-contrast CT (**a**) shows low attenuation in the right mesial temporal region (white arrows). DWI b1000 (**b**) and ADC map (**c**) confirm bilateral but asymmetrical restricted diffusion in the mesial temporal areas (white arrows and arrowhead). No haemorrhage is identified on T2* (**d**). There are signal abnormalities on FLAIR (**e**,**f**), most pronounced in the right mesial temporal lobe (white arrow), right insula (black arrowhead) and right frontal lobe (black arrow). MRI performed 2 months later (**g**,**h**) shows bilateral areas of encephalomalacia (white arrows and arrowhead), more pronounced on the right, despite treatment.

Destructive brain lesions involving cortex due to different types of insults, more commonly ischemia, trauma or infection, can result in porencephaly, encephalomalacia, ulegyria, gliosis and secondary epilepsy. These insults can occur anytime during life, from the antenatal period to late adulthood and the severity of clinical manifestation is usually related to the severity of volume loss, the location of the lesions near eloquent areas of the brain, as well the time when the insult occurred. Cerebrovascular disease is the most common cause of acquired epilepsy in western countries, affecting mostly older patients [87]. The presence of blood products in the brain, known as hemosiderosis, is considered highly epileptogenic [88], while an increased risk for seizures has been observed as a sequela of meningoencephalitis [87]. In addition to volume loss, CT demonstrates low attenuation lesions and MRI shows hyperintense lesions on T2WI and FLAIR, and hypointensity on T1WI, reflecting gliosis (Figure 10 and Figure 11). Hemosiderosis is depicted as low signal lining the brain surface on T2 GRE or SWI images.

### 4.8. Miscellaneous

**Figure 12:**
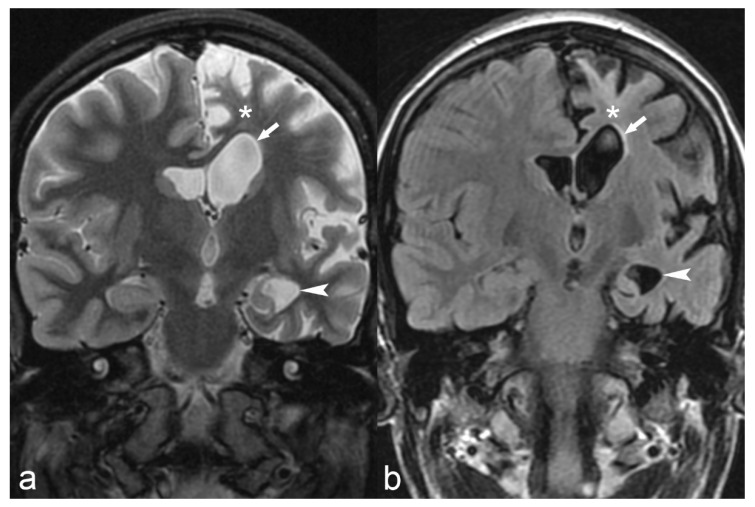
18-year-old female with medically refractory epilepsy. Coronal T2 (**a**) and FLAIR (**b**) images show hemispherical parenchymal volume loss and cortical thinning (asterisk) on the left side with resultant ex vacuo dilatation of the frontal (arrow) and temporal horns (arrowhead) of the left lateral ventricle, consistent with Rasmussen’s encephalitis.

Rasmussen’s encephalitis is a rare syndrome of unknown etiology, probably immune-mediated, often presenting with drug-resistant epilepsy at childhood. MRI in Rasmussen’s encephalitis reveals progressive atrophy of one of the cerebral hemispheres (Figure 12), usually beginning in the opercular region [22]. Often, the cortex and subcortical white matter present hyperintense signal on T2WI and FLAIR [22].

**Figure 13:**
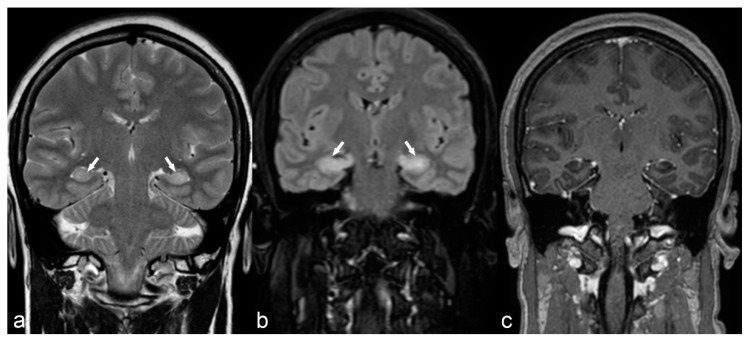
50-year-old woman. Symmetrical hyperintensity is identified on coronal T2WI (**a**) and FLAIR (**b**) in both hippocampi (arrows). No abnormal enhancement is seen on 3D T1 Gd (**c**). This is a case of autoimmune limbic encephalitis.

Auto-immune encephalitis is a less frequent cause of epilepsy characterized by high T2/FLAIR signal of the temporal lobe (including the hippocampus), insula and cingulum, usually with bilateral symmetrical or asymmetrical involvement (Figure 13).

**Figure 14:**
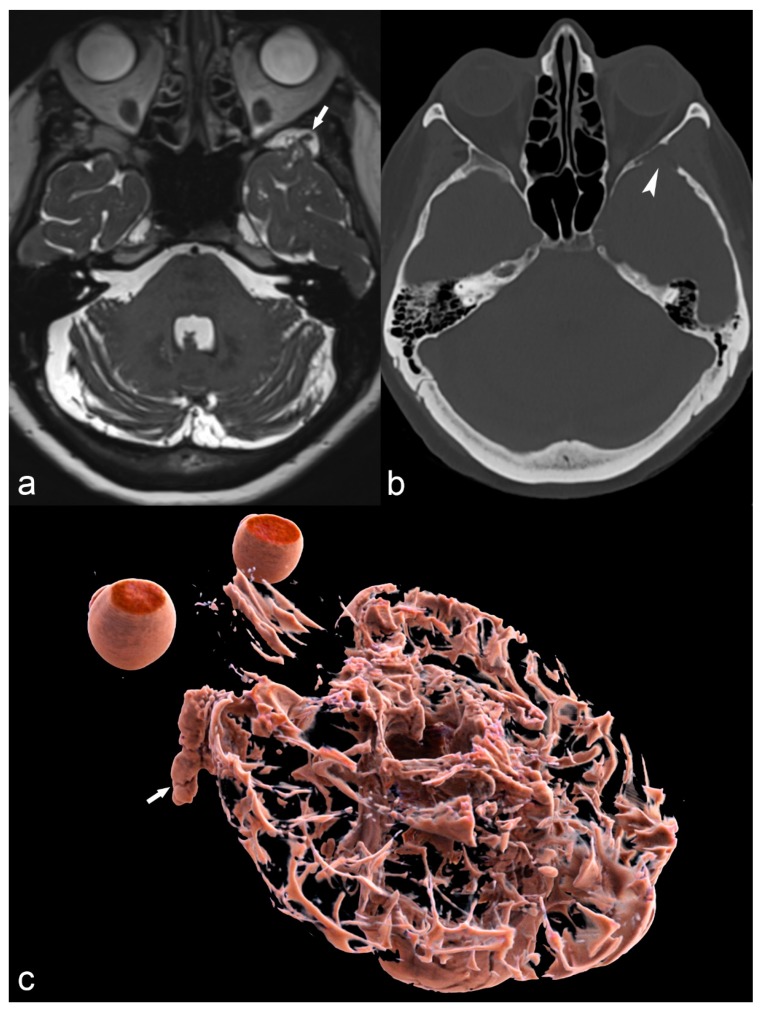
68-year-old woman with left temporal meningoencephalocele. Axial 3D high-ponderated T2 sequence (**a**) shows herniation of abnormal brain parenchyma, meninges and cerebrospinal fluid (arrow) into a defect of the greater wing of the sphenoid bone, best seen on CT in the bone window (arrowhead in (**b**)). This is further illustrated by a 3D cinematic rendering of the 3D T2 acquisition with a left posterior-anterior oblique vantage point (**c**), which demonstrates the full caudal extent of the meningoencephalocele within the sphenoid bone (arrow).

Meningoencephaloceles are probably an underreported cause of epilepsy, most commonly located in the middle cranial fossa. Smaller or larger bony defects of the greater wing of the sphenoid can result in encephaloceles, which cause irritation of the cortex and epilepsy, and their depiction sometimes requires a combination of thin-slice CT in the bony window and high-resolution coronal T2 MRI images (Figure 14).

## 5. Pitfalls and Artifacts

Although 3D FLAIR with thin slices can detect many epileptogenic lesions, 2D FLAIR optimized for contrast resolution (with thicker slices and therefore lower spatial resolution) may occasionally offer a better depiction of subtle T2 hyperintensities, like those seen in small FCD [23].

Another pitfall with 3D FLAIR is its inherent high signal in certain areas of the brain such as the extended limbic system, including the amygdala, hippocampus and cingulate gyrus [23], which can hinder the detection of HS or limbic encephalitis; a comparison with T2 images is necessary to overcome this problem. 3D FLAIR is also known to be associated with increased pulsation artifacts and radiologists should be aware of this in order to avoid overdiagnosis [23].

Attention should also be paid to the imaging characteristics of epileptogenic lesions in the immature brain of infants. Before approximately 6 months of age, the T2WI hyperintensity seen in the gray/white matter junction of adults with FCD corresponds to a hypointensity on T2W images [89] and the “transmantle sign” is not usually evident. For this reason, serial imaging is sometimes necessary for infants with refractory epilepsy.

## 6. Conclusions

Neuroimaging along with EEG are currently the most important tools for the diagnosis of medically refractory epilepsy in the pediatric and adult populations. Despite recent advances in neuroimaging methods, diagnosis of specific epileptogenic lesions remains challenging and a close correlation with semiology, EEG findings and imaging findings, both structural and metabolic, is mandatory. Combining these different techniques will allow surgery to be appropriately guided and offer these patients the chance of a seizure-free life, as well as allay some of the negative cognitive, psychological and socioeconomic consequences of drug-resistant epilepsy.

## Figures and Tables

**Table 1:** Proposed optimized 3T MRI protocol for epilepsy.

MRI Sequences	Additional Options	In Children Less 24 Months
**Axial DTI** (acquisition in 3 directions, ST: 2 mm, TE: 54, TR: 7400, AT: 4 min 13 s, matrix: 256 × 256, FOV: 230 × 230)	**3D T1 GRE** (ST: 0.9 mm, TE: 2.36, TR: 1930, AT: 3 min 33 s, matrix: 288 × 288, FOV: 250 × 250) and **axial T1 SE** (ST: 4 mm, TE:9.2 TR: 400, AT: 1 min 36 s, matrix: 243 × 320, FOV: 185 × 220) after Gadolinium injection **	**Coronal T1** (ST: 4 mm, other parameters depending on age)
**Axial T2 FSE** (ST: 4 mm, TE: 107, TR: 6610, AT: 2 min 58 s, matrix: 418 × 512, FOV: 199 × 220)		
**Axial SWI** (ST: 1.6 mm, TE: 20, TR: 28, AT: 3 min 40 s, matrix: 434 × 704, FOV: 193 × 220)		
**3D Sagittal FLAIR** (ST: 1 mm, TE: 386, TR: 5000, TA: 5 min 5 s, matrix 512 × 512, FOV: 256 × 256)		
Coronal T2 FSE * (ST: 3 mm, TE: 89, TR: 5210, AT: 2 min 31 s, matrix: 288 × 384, FOV: 189 × 189)		
**Sagittal 3D T1 GRE** (ST: 0.9 mm, TE: 2.36, TR: 1930, AT: 3 min 33 s, matrix: 288 × 288, FOV: 250 × 250)		
Sagittal 3D T1 MP2RGE *** (ST: 1 mm, TE: 2.9, TR: 5000, AT: 8 min 16 s, matrix: 240 × 256, FOV: 240 × 256)		

* perpendicular to the hippocampus, ** depending on initial findings and diagnostic suspicion *** either a 3D T1 GRE or a 3D T1 MP2RAGE can be performed. Abbreviations: ST: slice thickness, TE: time to echo, TR: repetition time, AT: acquisition time, FOV: field of view, FSE: fast spin echo, GRE: gradient-recalled echo, SE: spin echo.
